# Secreted Frizzled‐Related Protein 2 Promotes Osteogenic Differentiation and Bone Regeneration in Perthes Disease When Targeted by miR‐106a‐5p

**DOI:** 10.1111/jcmm.70804

**Published:** 2025-09-21

**Authors:** Tianjiu Zhang, Jiafei Yang, Song Yu

**Affiliations:** ^1^ Department of Pediatric Surgery, Guizhou Children's Hospital Affiliated Hospital of Zunyi Medical University Zunyi China; ^2^ School of Clinical Medicine Guizhou Medical University Guiyang China

**Keywords:** bone regeneration, miR‐106a‐5p, osteogenic differentiation, Perthes disease, secreted frizzled‐related protein 2

## Abstract

Although the pathogenesis of Perthes disease remains unclear, the mechanism of bone regeneration in the defective femoral head is an area of particular interest. Improving understanding of the underlying mechanisms is essential for the development of an effective treatment for this condition. This study explored the roles of secreted frizzled‐related protein 2 (SFRP2) and micro ribonucleic acid (miR)‐106a‐5p in the regulation of osteogenic differentiation (in vitro) and bone regeneration (in vivo). Cells were transfected with plasmids carrying the genes encoding SFRP2 and miR‐106a‐5p. Cell proliferation and apoptosis were evaluated using 5‐ethynyl‐2′‐deoxyuridine staining, flow cytometry and the terminal deoxynucleotidyl transferase dUTP nick end‐labelling assay. Osteogenic differentiation was identified using alkaline phosphatase and alizarin red S staining. Reverse transcription quantitative polymerase chain reaction and western blot were used to assess messenger ribonucleic acid and protein levels. The dual‐luciferase reporter gene assay was used to confirm the targeting relationship between miR‐106a‐5p and SFRP2. Changes in bone structure were evaluated by morphological observation, micro‐computed tomography and alkaline phosphatase staining. The findings showed that SFRP2 significantly increased cell proliferation and osteogenic differentiation and inhibited apoptosis of bone marrow mesenchymal stem and MC3T3‐E1 cells. It upregulated the expression of PCNA, Bcl‐2, ALP, Col1a1, Runx2, Osterix, Wnt3a, β‐catenin, LRP5 and LRP6, and downregulated that of Bax. Negative regulation of SFRP2 by miR‐106a‐5p (via the Wnt/β‐catenin pathway) could rescue the influence of the latter; this showed that SFRP2 is a target gene of miR‐106a‐5p.In conclusion, miR‐106a‐5p/SFRP2 may play a crucial role in bone regeneration in the defective femoral head and may be a potential therapeutic target in Perthes disease.

## 1 Introduction

Perthes disease is characterised by ischemic necrosis of the proximal femoral epiphysis in children. The condition, which results from a decrease in blood supply to the femoral head, commonly leads to malformation and early onset degenerative arthritis of the hip joint; this is associated with a high rate of disability [1, 2]. In the absence of any effective treatment, the main goals include restoration of the spherical structure of the femoral head and improvement of the consistency of both the femoral head and acetabulum [3, 4]. As the pathophysiology of Perthes disease remains unclear [2, 5], it is important to understand the mechanism of bone regeneration and accelerate bone repair. In addition to reversing the femoral deformity, this may help identify the best treatment for the condition.

Secreted frizzled‐related protein 2 (SFRP2) is vital for the growth and development of bone and cartilage [6, 7]. Studies have confirmed that SFRP2 expression is significantly up‐regulated during limb development in children. The lack of SFRP2 leads to reduced proliferation and delayed differentiation of chondrocytes and results in syndactylism and brachydactylism [8, 9]. In their study, de Castro et al. found that SFRP2 plays an important role in self‐renewal, recruitment, and differentiation of adult skeletal stem cells (SSCs) during bone healing. They also noted that knockout of the gene encoding SFRP2 obviously inhibited osteogenic differentiation of bone marrow stromal cells (BMSCs) and SSCs [10]. These findings indicate that SFRP2 can promote osteogenic differentiation of stem cells. In our study on an immature rabbit model of Perthes disease, ribonucleic acid sequencing (RNA‐seq) also showed a significant increase in SFRP2 expression in the femoral head during the repair phase [11].

MicroRNAs (miRNAs) are short endogenous noncoding RNAs that regulate various biological processes via post‐transcriptional repression of specific target mRNAs [12]. Available evidence suggests that miR‐106a‐5p plays an important role in regulating the development of osteocartilage and the maintenance of bone homeostasis [13, 14]. In their study, Hui et al. found that miR‐106a‐5p was significantly upregulated in BMSCs that were derived from adolescent patients with idiopathic scoliosis [15]; this was believed to be related to the resulting loss in bone mass. These findings indicate that miR‐106a‐5p is able to inhibit the differentiation of osteogenic precursor cells. Our experimental data have also shown that miR‐106a‐5p may have an inhibitory effect on osteogenesis [11]. In addition, the TargetScan database predicted a direct binding site between miR‐106a‐5p and SFRP2 mRNA‐3′UTR. It is therefore likely that the miR‐106a‐5p/SFRP2 axis regulates osteogenesis and bone regeneration.

Based on the possible interaction between miR‐106a‐5p and SFRP2, we hypothesised that SFRP2 may promote osteogenic differentiation of osteoblast precursors and accelerate bone regeneration in the defective femoral head on being targeted by miR‐106a‐5p (via the Wnt/β‐catenin pathway). The miR‐106a‐5p/SFRP2 axis was therefore considered to be a potential target for Perthes disease.

## 2 Material and Methods

### 2.1 Isolation and Culture of Primary Rabbit BMSCs

The procedures in this study were approved by the Medical Ethics Committee of the Guizhou Medical University, Guizhou, China (approval number: 2201637). The isolation and culture of primary rabbit BMSCs have been described previously [16]. In summary, bone marrow was pumped and flushed out from the metaphysis of the femur and tibia of eight‐week‐old rabbits, and density‐gradient centrifugation was employed. The obtained BMSCs were then plated in complete Dulbecco's Modified Eagle Medium (DMEM) (BasalMedia, China) supplemented with 10% fetal bovine serum (Gibco, USA) and 1% penicillin/streptomycin (BasalMedia, China); they were then incubated at 37°C in 5% carbon dioxide and the medium was refreshed every 3 days. Morphological identification of BMSCs was performed under a light microscope, and the cell phenotype was verified by flow cytometry; cells from passage three were used for the subsequent experiments.

### 2.2 Cell Transfection

Cell transfection was performed in accordance with the manufacturer's protocol. The BMSCs and MC3T3‐E1 cells were seeded onto a 24‐well plate at a density of 1 × 10^5^ cells/mL. On attainment of 60%–80% cell confluence, the plasmids were transfected using Lipofectamine 2000 reagent (Thermo Fisher, USA) according to the manufacturer's instructions. The overexpression SFRP2 plasmid vectors (oe‐SFRP2), blank negative control plasmid vector (NC), SFRP2‐targeting short hairpin RNA plasmid vectors (sh‐SFRP2), miR‐106a‐5p mimics (5′‐GAUGGACGUGACAUUCGUGAA AA‐3′), and miR‐106a‐5p inhibitor (5′‐CAAACACCUUACACACC AGGUAG‐3′) were synthesised by Guangzhou RiboBio Biotechnology Co. Ltd. In summary, 0.8–1.0 μg each of oe‐SFRP2, sh‐SFRP2, miR‐106a‐5p mimics, and inhibitor were diluted in 50 μL DMEM (Thermo Fisher, USA); 1–3 μL of Lipofectamine 2000 reagent was also diluted in 50 μL DMEM and maintained at room temperature for 4 h. Diluted deoxyribonucleic acid and Lipofectamine 2000 reagent were admixed and incubated at room temperature for 4 h; this mixture was then added to the cells and incubated for 2 h. Following further incubation in DMEM (at 37°C with 5% carbon dioxide for 48 h), the transfection efficiency was assessed using reverse transcription quantitative polymerase chain reaction (RT‐qPCR) and western blotting.

### 2.3 Osteogenic Differentiation

For induction of osteogenic differentiation, the BMSCs and MC3T3‐E1 cells (Ruyao Biotech, China) were seeded onto a 12‐well plate with osteoblast‐specific induction medium that contained 0.2 mM ascorbic acid, 100 nM dexamethasone, and 10 mM β‐glycerophosphate; the culture medium was replaced every 3 days. The cells were then harvested and analysed on the indicated days.

### 2.4 5‐Ethynyl 2′‐Deoxyuridine (EdU) Staining

EdU staining was performed using the BeyoClickEdU‐594 detection kit (Beyotime Biotech, China). The cells were seeded in a six‐well plate (5 × 10^5^ cells/well) for 24 h and then incubated with 100 μM EdU for 2 h. These cells were then immobilised with 4% paraformaldehyde for 15 min and permeabilised with 0.5% Triton X‐100 for 15 min. Nuclear staining was performed with Hoechst 33342, after washing with methanol and phosphate buffered saline (PBS) for 20 min. After staining, the cell numbers were counted per field and analysed quantitatively using IPP 6.0 software.

### 2.5 Flow Cytometry

The cells were cultured in a six‐well plate (1 × 10^6^ cells/well) and incubated in osteogenic induction medium for 7 days. After adjusting the cell density to 5 × 10^6^/mL, the cells were washed with PBS; 5 μL of Annexin V/FITC and 5 μL of propidium iodide were then added directly as per the instructions of the Annexin V‐FITC/PI apoptosis detection kit (Solarbio, China). The cells were then gently vortexed and incubated at room temperature in the dark for 15 min before performing flow cytometry.

### 2.6 Terminal Deoxynucleotidyl Transferase dUTP Nick End‐Labelling (TUNEL) Assay

The TUNEL assay was performed as per the instructions of the TUNEL cell apoptosis detection kit (Servicebio, China). The cells were seeded onto a six‐well plate (1 × 10^6^ cells/well); following osteogenic induction culture for 7 days, they were fixed in 4% paraformaldehyde for 20 min. The cells were then washed with PBS for 15 min and incubated with 0.2% Triton X‐100 for 5 min. After washing thrice with PBS, the cells were incubated with 56 μL of the reaction solution (in which the enzyme and label solutions were combined) at 37°C for 1 h. They were then washed with PBS for 20 min at room temperature and incubated at 37°C for 30 min with 100 μL of the haematoxylin (Servicebio, China). A coverslip was placed and images were captured under a fluorescence microscope (Leica, Germany). At least 100 cells were counted in 10 random fields, and the percentage of positive apoptotic cells was calculated.

### 2.7 Alkaline Phosphatase (ALP) and Alizarin Red S (ARS) Staining

ALP staining was performed using the ALP staining kit (Beyotime Biotech, China) according to the manufacturer's recommendations. The cells were fixed with 4% paraformaldehyde at room temperature for 20 min after osteogenic induction for 7 days. They were then washed with PBS, and the stained cells on the plates were photographed; the absorbance value at 405 nm was measured using an enzyme‐labelled instrument (Molecular, USA) to quantify ALP activity. For ARS staining, the cells were fixed with 4% paraformaldehyde for 20 min after osteogenic induction for 28 days. The mineralisation tubercle was then incubated with 40 mM alizarin red S dye (Macklin, China) at 37°C for 20 min. The stained cells on the plates were photographed, and the stain was dissolved in 10% cetylpyridinium chloride. The absorbance of the extracted solution was then quantified using an enzyme‐labelled instrument (Molecular, USA) at 562 nm.

### 2.8 Dual‐Luciferase Reporter Gene Assay

The interactive relationship between miR‐106a‐5p and SFRP2 was confirmed using the dual‐luciferase reporter gene assay. The TargetScan database (http://www.targetscan.org) was used to predict the putative binding site of miR‐106a‐5p at SFRP2 3′UTR. The assay was performed using the Dual‐glo Dual Luciferase Test Kit (Promega, USA) according to the manufacturer's recommendations. The wild‐type (WT) and mutant‐type (MUT) SFRP2 3′UTR vectors were constructed by RiboBio Biotechnology Co. Ltd. (Guangzhou, China). The host 293T cells were seeded and cultured in 96‐well plates with the indicated reporter construct and a Renilla luciferase plasmid. Luciferase activity was measured at 24 h after transfection using a fluorescence spectrophotometer (Promega, USA) according to the manufacturer's instructions. The relative transcriptional activity was normalised to the corresponding vehicle control value.

### 2.9 RT‐qPCR

Total RNA was extracted from the cells or bone tissue using TRIzol reagent (Thermo Fisher, USA) according to the manufacturer's instructions. The RNA concentrations and optical density (1.8–2.1) were measured using a spectrophotometer (Thermo Fisher, USA), and RNA integrity was assessed by 1% E‐GelEX agarose gel electrophoresis (Thermo Fisher, USA). Total RNA was reverse transcribed into complementary deoxyribonucleic acid using the PrimeScript RT reagent Kit with gDNA Eraser (Takara, Japan). The NovoStart SYBR qPCR SuperMix Plus kit (Yeasen, China) was used for qPCR; the PCR primer sequences used in this study are listed in Table 1. Relative RNA was normalised to endogenous β‐actin or U6, and qPCR was performed using the 7500 Real‐Time PCR system using SYBR green (Thermo Fisher, USA) under the following thermocycling conditions (amplification conditions): 95°C for 5 min, 95°C for 10 s, 60°C for 30 s and 72°C for 30 s, for a total of 35 cycles. The 2−ΔΔCq method was used to determine the relative fold change in expression between the experimental and control groups.

**TABLE 1:** RNA primer sequences.

Genes	Sequence (5′‐3′)
β‐Actin	
Forward	CATTGCTGACAGGATGCAGAAGG
Reverse	TGCTGGAAGGTGGACAGTGAGG
SFRP2	
Forward	AGGACAACGACCTCTGCATC
Reverse	AGCCACAGCACGGATTTCTT
GAPDH	
Forward	CATCACTGCCACCCAGAAGACTG
Reverse	ATGCCAGTGAGCTTCCCGTTCAG
PCNA	
Forward	CAAGTGGAGAGCTTGGCAATGG
Reverse	GCAAACGTTAGGTGAACAGGCTC
Bax	
Forward	AGGATGCGTCCACCAAGAAGCT
Reverse	TCCGTGTCCACGTCAGCAATCA
Bcl‐2	
Forward	CCTGTGGATGACTGAGTACCTG
Reverse	AGCCAGGAGAAATCAAACAGAGG
Caspase3	
Forward	GGAGTCTGACTGGAAAGCCGAA
Reverse	CTTCTGGCAAGCCATCTCCTCA
Wnt3a	
Forward	AACTGCACCACCGTCAGCAACA
Reverse	AGCGTGTCACTGCGAAAGCTAC
Catenin (CTNNB1)	
Forward	GTTCGCCTTCATTATGGACTGCC
Reverse	ATAGCACCCTGTTCCCGCAAAG
LRP5	
Forward	CCTCACCATTGATTATGCCGACC
Reverse	GATCGTCAGCTATCACCATGCG
ALPL	
Forward	CCAGAAAGACACCTTGACTGTGG
Reverse	TCTTGTCCGTGTCGCTCACCAT
Col1a1	
Forward	AGCCTCTCCATCTTTGCCAGCA
Reverse	CAGAAGGACCTTGTTTGCCAGG
Runx2	
Forward	CCTGAACTCTGCACCAAGTCCT
Reverse	TCATCTGGCTCAGATAGGAGGG
Osterix (SP7)	
Forward	GGCTTTTCTGCGGCAAGAGGTT
Reverse	CGCTGATGTTTGCTCAAGTGGTC
miR‐106a‐5p	
Forward	UAAAGUGCUGACAGUGCAGAUAG
Reverse	AGTGCAGGGTCCGAGGTATT
U6	
Forward	GCCATACCACCCTGAACG
Reverse	GGTATTCCCAGGCGGTCT

### 2.10 Western Blot

Cells or bone tissue were lysed using radioimmunoprecipitation assay buffer with a complete protease inhibitor (Beyotime Biotech, China). Following quantification using a bicinchoninic acid protein assay, the samples were separated using sodium dodecyl sulfate‐polyacrylamide gel electrophoresis and transferred onto polyvinylidene fluoride membranes (Bio‐Rad, USA) as per the standard process. The membranes were then blocked with nonfat milk and incubated overnight at 4°C with anti‐SFRP2 (Huabio, China), anti‐PCNA (Huabio, China), anti‐Bax (Huabio, China), anti‐Bcl‐2 (Huabio, China), anti‐Osterix (Huabio, China), anti‐Runx2 (Huabio, China), anti‐Col1a1 (Abcam), anti‐ALP (Abcam), anti‐Wnt3a (Huabio, China), anti‐β‐catenin (Huabio, China), anti‐LRP5 (Abcam) and anti‐LRP6 (Abcam) antibodies. Image J software was used for quantitative analysis of the blots.

### 2.11 Animal Studies

Eight‐week‐old New Zealand White rabbits were provided by Jinan Jinfeng Experimental Animal Co. Ltd. (number: SCXK20180006). They were bred adaptively for 7 days at a room temperature of 25°C; relative humidity was maintained at 55%. They were allowed free access to water and were maintained in 12‐h light/dark cycles. All procedures in this study complied with the recommendations of the National Institutes of Health Guide for the Care and Use of Laboratory Animals (2020/3rd ed). Adeno‐associated virus vectors containing AAV‐zsGreen‐SFRP2 (Ad‐SFRP2) and miR‐106a‐5p agomir were synthesised by Guangzhou RiboBio Co. Ltd. These rabbits were randomly divided into five groups, namely, the control (normal), model, miR‐106a‐5p agomir, Ad‐SFRP2, and Ad‐SFRP2 + miR‐106a‐5p agomir groups; five rabbits were included in each group. In summary, the rabbits were anaesthetised using intraperitoneal pentobarbital sodium. The rabbit model of Perthes disease was then produced by incising the ligamentum teres of the femoral head and ligating the femoral neck with an elastic non‐absorbable suture; this obstructed blood flow to the epiphysis. Ad‐SFRP2 (1 × 108 GC) and miR‐106a‐5p agomir (20 nmol) were injected into the femoral head 2 weeks after surgery (Figure S1); the same volumes of saline were used in the control and model groups. The rabbits were then euthanised at 8 weeks after surgery, and the femoral heads were harvested for subsequent experiments.

### 2.12 Macroscopic Morphological Observation

The femoral heads were evaluated at the eighth week after surgery based on visual morphology. They were assessed for size, spherical integrity, and presence of collapse; the smoothness, colour, and thickness of the articular cartilage were also evaluated.

### 2.13 Micro‐Computed Tomography (Micro‐CT) Analysis

The femoral head was subjected to fixation in 4% paraformaldehyde for 2 days prior to analysis. A micro‐CT imaging system (Zhongke Kaisheng, China) with a voltage of 70 kV and a resolution of 8 mm was used for evaluation. The following parameters of the trabecular bone were analysed: bone volume/total volume, bone mineral density (in mg/cc), bone mineral content (in mg), trabecular number (per mm), trabecular thickness (in mm), trabecular space (in mm) and connectivity density (per mm^3^).

### 2.14 Histological and Immunohistochemical Analysis and ALP Staining

Following micro‐CT analysis, the femoral head was fixed in 4% paraformaldehyde, decalcified in 10% ethylenediaminetetraacetic acid and washed in running water. The decalcified tissue was dehydrated, diaphanised, and embedded in paraffin before sectioning into 4‐μm slices. ALP staining was performed as described in Section 2.7, and absorbance values were measured for quantification. The proportion of empty bone lacunae was counted using haematoxylin and eosin staining, and SFRP2‐positive cells were identified using immunohistochemical staining with an anti‐SFRP2 antibody (Huabio, China).

### 2.15 Statistical Analysis

Statistical analysis was performed using SPSS software 23.0 (IBM, USA). Quantitative data were presented as means ± standard deviation (SD), and the Kolmogorov–Smirnov test was used to assess normality of distribution. The variances among different groups were compared using the *F* test, and the unpaired Student's *t*‐ or Mann–Whitney *U* tests were used for intergroup comparison. In the case of three or more groups, the Tukey's post hoc test was performed after one‐way analysis of variance; *p* < 0.05 was considered significant.

## 3 Results

### 3.1 SFRP2 Promoted Proliferation and Inhibited Apoptosis in BMSCs and MC3T3‐E1 Cells

**FIGURE 1:**
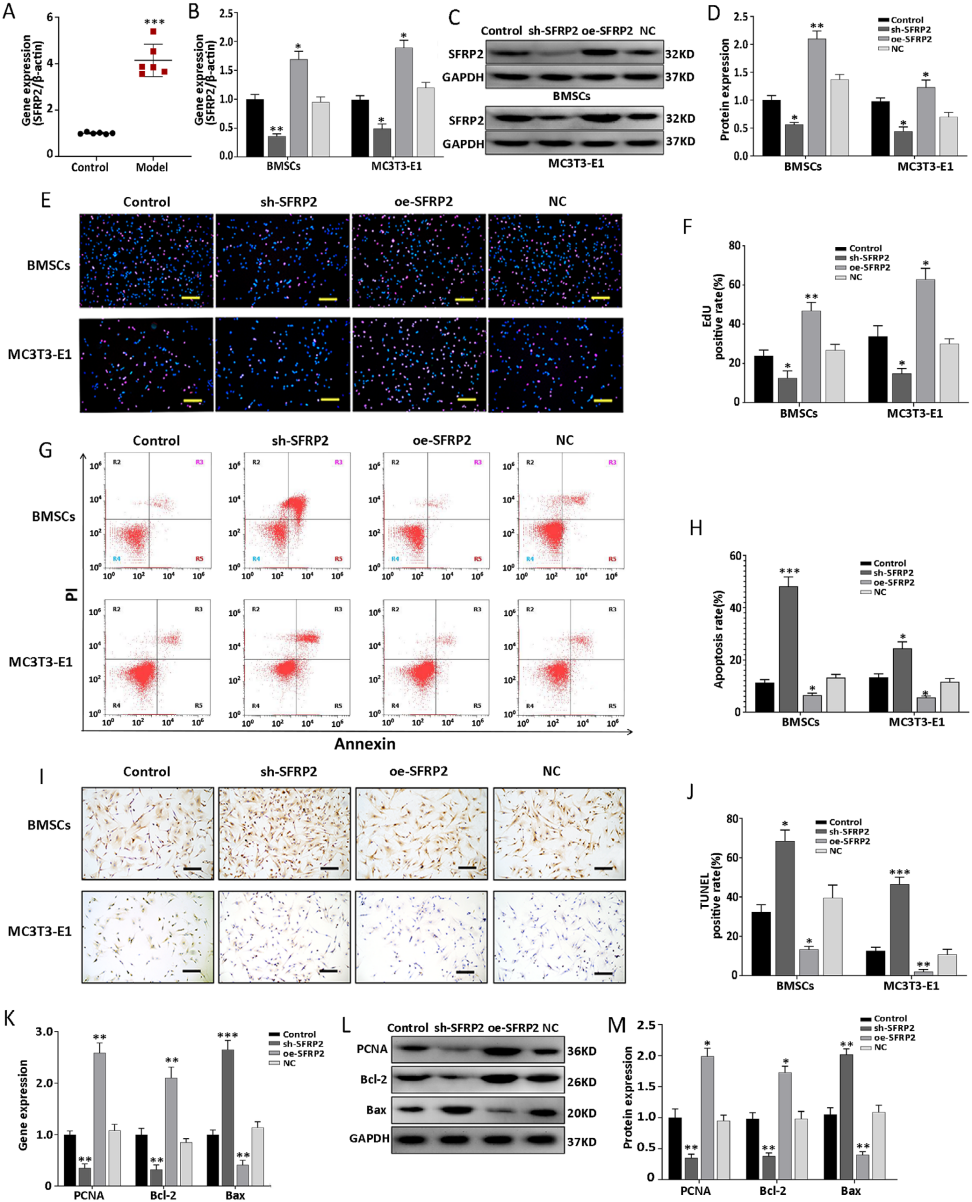
SFRP2 promotes proliferation and inhibits apoptosis in BMSCs and MC3T3‐E1 cells. (A) The in vivo mRNA expression profile of SFRP2 in the control and model groups, as tested using qPCR. (B) The mRNA expression profile of SFRP2, as tested in the oe‐SFRP2, NC, sh‐SFRP2 and control groups. (C, D) Expression of SFRP2 protein, measured by western blot and analysed quantitatively. (E, F) EdU staining showing and quantifying cell proliferation. (G, H) Flow cytometry detecting and quantifying cell apoptosis. (I, J) TUNEL staining showing and quantifying cell apoptosis. (K) mRNA expression profile of PCNA, Bcl‐2, Bax. (L, M) Protein expression profile of PCNA, Bcl‐2, Bax (and quantitative analysis results). Scale bars = 100 μM. Data are shown as means ± SD for three independent experiments. *p < 0.05, **p < 0.01, ***p < 0.001 versus control group. Model group: Defective femoral head in immature rabbits with Perthes disease.

RT‐qPCR was performed to verify the results of RNA sequencing; it confirmed upregulation of SFRP2 expression in the defective femoral head of the rabbit model (model group) (Figure 1A). The BMSCs and MC3T3‐E1 were transfected with the respective lentiviral vectors to further investigate the regulatory effect of SFRP2 on cell proliferation and apoptosis. Transfection efficiency was confirmed using RT‐qPCR and western blotting (Figure 1B–D). EdU staining showed a significant increase in cell proliferation (Figure 1E,F); the expression of PCNA was also found to be considerably higher in the oe‐SFRP2 group than in the NC group (Figure 1K–M). However, flow cytometry and the TUNEL assay showed remarkable suppression of cell apoptosis (Figure 1G–J); the oe‐SFRP2 group demonstrated downregulation of Bax expression and upregulation of Bcl‐2 expression (Figure 1K–M). However, the sh‐SFRP2 group showed the opposite findings (Figure 1). These results suggested that SFRP2 can promote proliferation and inhibit apoptosis of osteoblast progenitor cells.

### 3.2 SFRP2 Enhanced Osteogenic Differentiation of BMSCs and MC3T3‐E1 Cells via the Wnt/β‐Catenin Pathway

**FIGURE 2:**
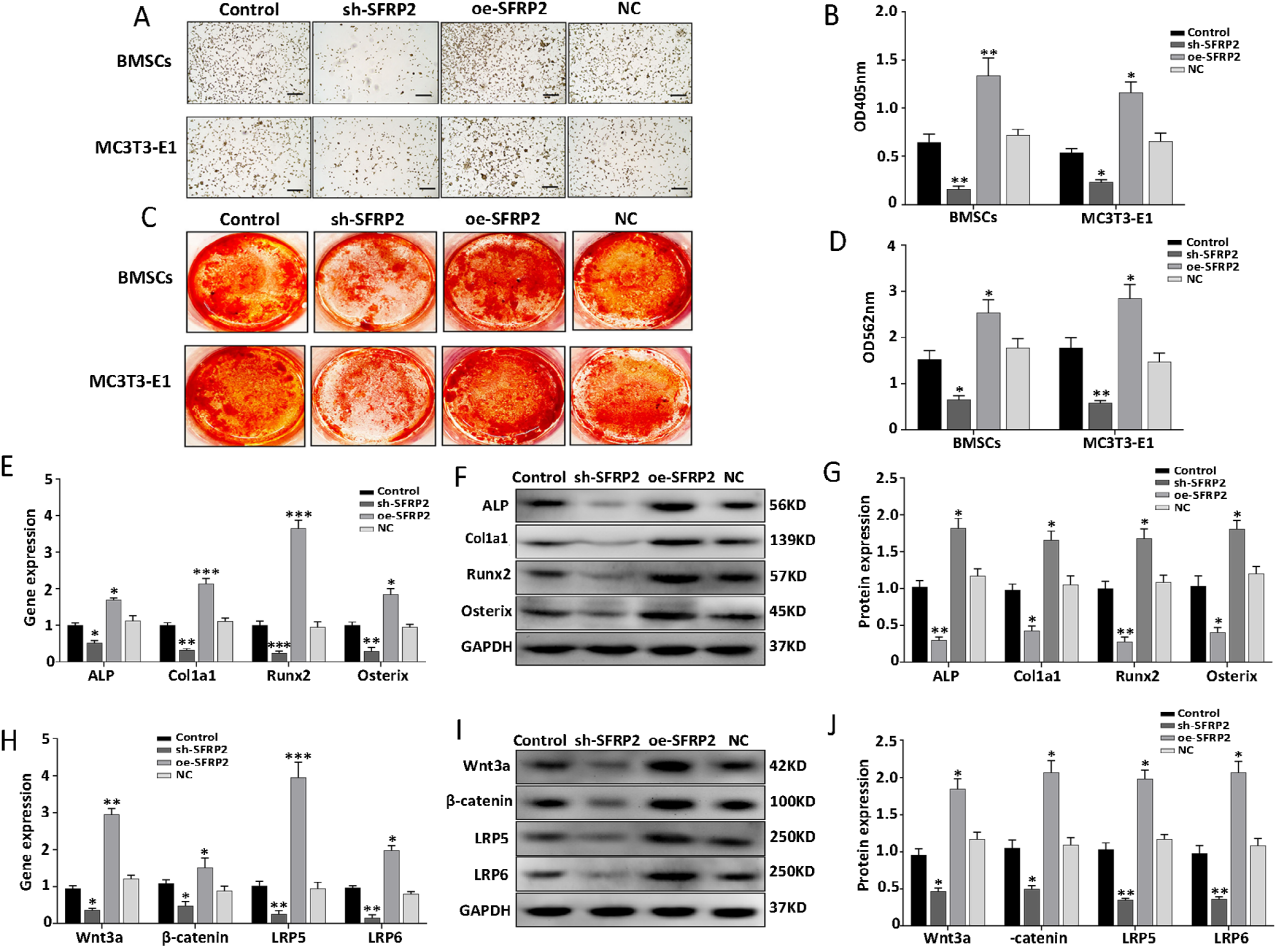
SFRP2 enhances osteogenic differentiation via Wnt/β‐catenin pathway in BMSCs and MC3T3‐E1 cells. (A, B) ALP staining and quantification. (C, D) ARS staining and quantification. (E) mRNA expression profile of osteogenic marker genes. (F, G) Protein expression of osteogenic marker genes (and quantitative analysis results). (H) mRNA expression profile of key genes in the Wnt/β‐catenin pathway. (I, J) Protein expression profile of the Wnt/β‐catenin pathway and quantitative analysis. Scale bars = 100 μM. Data are shown as means ± SD for three independent experiments. *p < 0.05; **p < 0.01; ***p < 0.001 versus the control group.

The effect of SFRP2 on osteogenic differentiation of BMSCs and MC3T3‐E1 cells was then evaluated. As shown in Figure 2A–D, the intensity of ALP staining was the highest on day 7 of osteogenic differentiation; the activity was also the strongest at this time. ARS staining on day 28 also showed a higher number of calcified nodules and higher staining intensity in the oe‐SFRP2 group than in the other groups. In addition, RT‐qPCR and western blot showed a significant increase in the expression of osteogenic molecules (namely, ALP, Col1a1, Runx2 and Osterix) in the oe‐SFRP2 group (Figure 2E–G). To explore whether SFRP2 influenced the process of osteogenesis via the canonical Wnt/β‐catenin pathway (which has been proven to be vital for osteogenic differentiation), the relevant biomarkers were evaluated using RT‐qPCR and western blot. The findings indicated that the expression of Wnt3a, β‐catenin, LRP5, and LRP6 had increased significantly in the oe‐SFRP2 group (Figure 2H–J). However, the sh‐SFRP2 group showed the opposite results (Figure 2). These findings indicate that SFRP2 enhances osteogenic differentiation of osteoblast precursors via the Wnt/β‐catenin pathway.

### 3.3 SFRP2, Which Is Negatively Regulated by miR‐106a‐5p, Is a Direct Target Gene of miR‐106a‐5p

**FIGURE 3:**
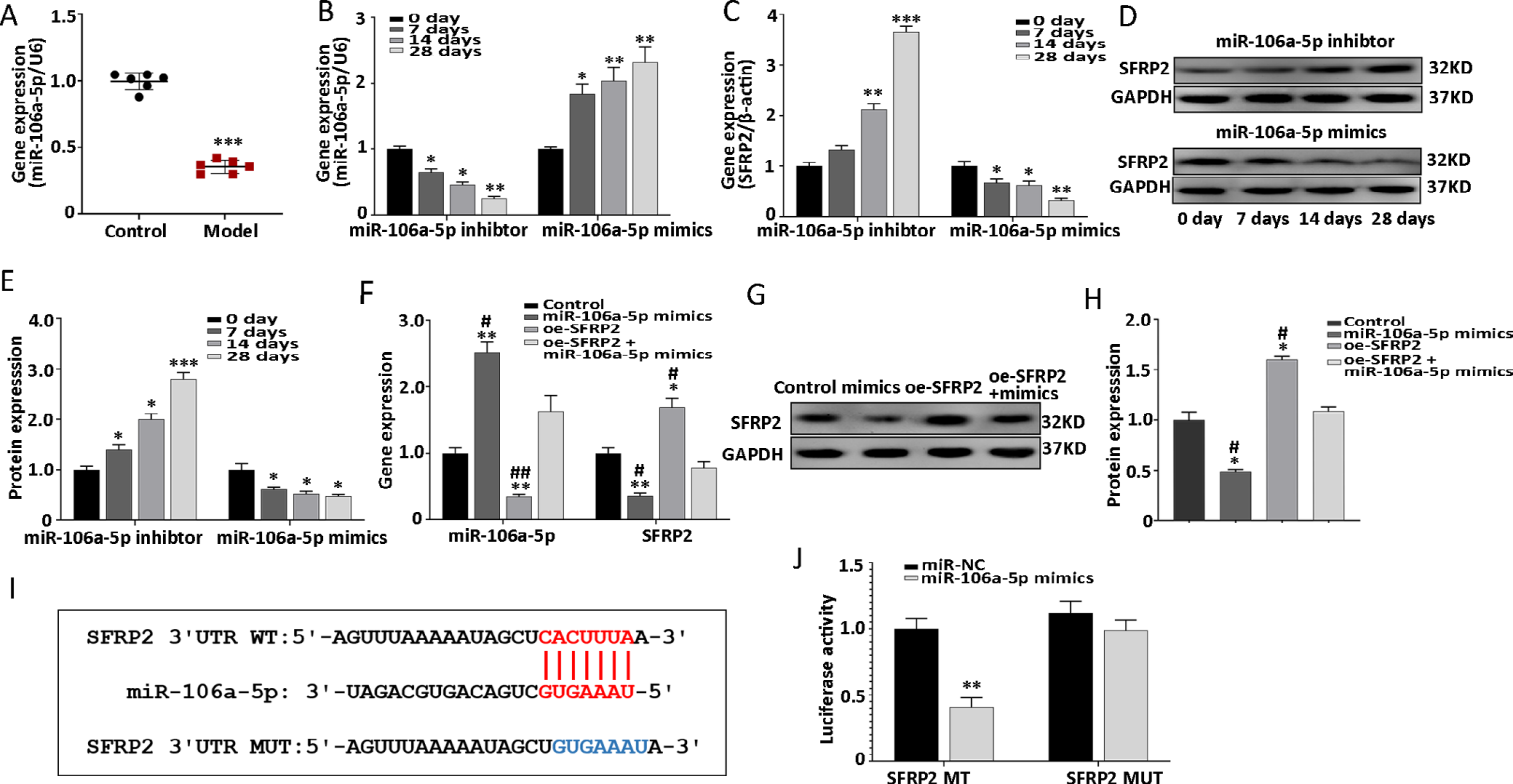
SFRP2 is a direct target of miR‐106a‐5p. (A) The in vivo expression of miR‐106a‐5p, as tested in the control and model groups using RT‐qPCR. (B, C) Gene expression of miR‐106a‐5p and SFRP2 in the miR‐106a‐5p inhibitor and mimics groups at different time points. (D, E) Protein expression profile of SFRP2 (and quantitative analysis results). (F) Gene expression profile of miR‐106a‐5p and SFRP2 in the control, miR‐106a‐5p mimics, oe‐SFRP2, and miR‐106a‐5p mimics + oe‐SFRP2 groups. (G, H) Protein expression profile of SFRP2 (and quantitative analysis results). (I) Binding site between SFRP2 3′UTR and miR‐106a‐5p. (J) Luciferase activity of SFRP2‐WT and SFRP2‐MUT after transfection with miR‐NC or miR‐106a‐5p mimics. Data are shown as means ± SD for three independent experiments. *p < 0.05, **p < 0.01, ***p < 0.001 versus day 0 or control group; ^#^ *p* < 0.05, ^##^ *p* < 0.01, versus miR‐106a‐5p mimics + oe‐SFRP2 group. Model group: The defective femoral head of immature rabbits with Perthes disease.

To verify the results of RNA sequencing, RT‐qPCR was used to confirm downregulation of miR‐106a‐5p expression in the defective femoral head (model group) (Figure 3A). In addition, the BMSCs were transduced with the miR‐106a‐5p mimics and miR‐106a‐5p inhibitor to further explore the regulatory effect of miR‐106a‐5p on SFRP2. The expression profiles of miR‐106a‐5p and SFRP2 were then evaluated using RT‐qPCR and western blot on days 0, 7, 14 and 28 of osteogenic differentiation. The findings indicated significant downregulation of SFRP2 expression in the miR‐106a‐5p mimics group, and remarkable upregulation in the miR‐106a‐5p inhibitor group in a time‐dependent manner (Figure 3B–E). To further confirm the interaction between miR‐106a‐5p and SFRP2, miR‐106a‐5p mimics were co‐transduced with oe‐SFRP2. The miR‐106a‐5p mimics group demonstrated a marked reduction in SFRP2 expression at 24 h after transfection; in addition, the miR‐106a‐5p mimics + oe‐SFRP2 group showed that the regulatory effect of miR‐106a‐5p could be rescued by SFRP2 (Figure 3F–H). A dual luciferase reporter assay was then performed to further elucidate the interaction between miR‐106a‐5p and SFRP2. The TargetScan database was also used to predict the binding site between miR‐106a‐5p and SFRP2 3′UTR (Figure 3I). The assay showed that miR‐106a‐5p mimics suppressed SFRP2 3′UTR WT luciferase reporter activity, but not that of the SFRP2 3′UTR MUT reporter (Figure 3J). In conjunction, these results demonstrated that SFRP2, which is negatively regulated by miR‐106a‐5p, is a direct target gene of miR‐106a‐5p.

### 3.4 miR‐106a‐5p Negatively Regulated SFRP2 to Inhibit Proliferation and Osteogenic Differentiation, and Promote Cell Apoptosis, via the Wnt/β‐Catenin Pathway

**FIGURE 4:**
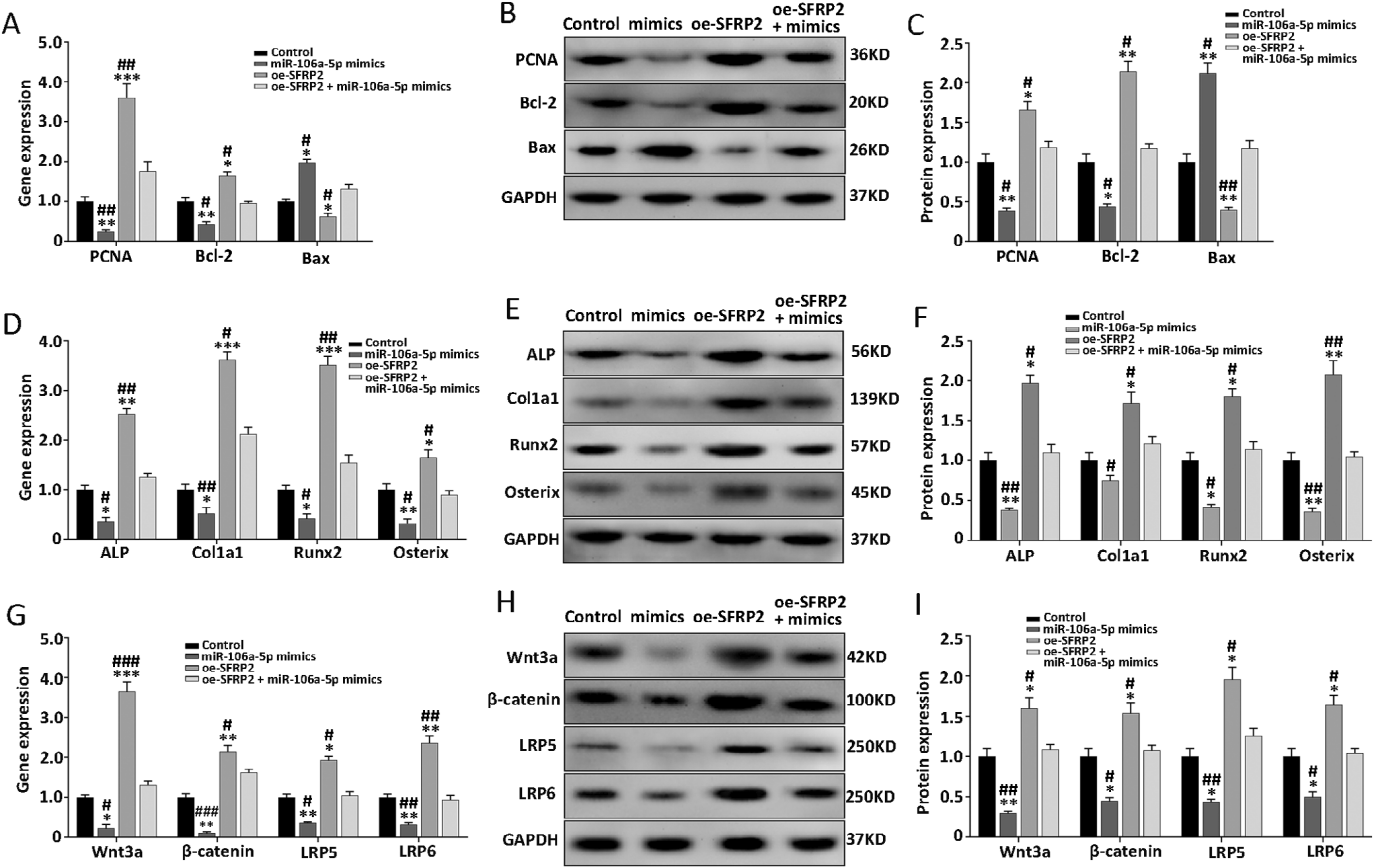
miR‐106a‐5p negatively regulates SFRP2 to inhibit proliferation and osteogenic differentiation and promote apoptosis via the Wnt/β‐catenin pathway in BMSCs. (A) mRNA expression profile of PCNA, Bcl‐2, Bax in the control, miR‐106a‐5p mimics, oe‐SFRP2, and miR‐106a‐5p mimics + oe‐SFRP2 groups. (B, C) Protein expression profile of PCNA, Bcl‐2, Bax (and quantitative analysis results). (D) mRNA expression profile of osteogenic marker genes. (E, F) Protein expression profile of osteogenic marker genes (and quantitative analysis results). (G) mRNA expression profile of key genes in the Wnt/β‐catenin pathway. (H, I) Protein expression profile of the Wnt/β‐catenin pathway (and quantitative analysis results). Results are shown as means ± SD for three independent experiments. *p < 0.05, **p < 0.01, ***p < 0.001 versus the control group; ^#^ *p* < 0.05, ^##^ *p* < 0.01, ^###^ *p* < 0.001 versus miR‐106a‐5p mimics + oe‐SFRP2 group.

To further confirm the role of SFRP2 as a mediator in the regulation of cell proliferation, apoptosis, and osteogenic differentiation by miR‐106a‐5p, the BMSCs were co‐transfected with oe‐SFRP2, miR‐106a‐5p mimics, and their controls; they were then cultured in an osteogenic medium. The findings from RT‐qPCR and western blot showed that the expression of PCNA, Bcl‐2, ALP, Col1a1, Runx2, and Osterix had markedly decreased in the miR‐106a‐5p mimics group, while that of Bax had increased significantly; however, the oe‐SFRP2 group showed opposite findings. In addition, the regulatory effect of miR‐106a‐5p could be rescued by SFRP2 in the miR‐106a‐5p mimics + oe‐SFRP2 group (Figure 4).

### 3.5 SFRP2 Accelerated Bone Regeneration and Reduced Femoral Head Deformity in the Rabbit Model

**FIGURE 5:**
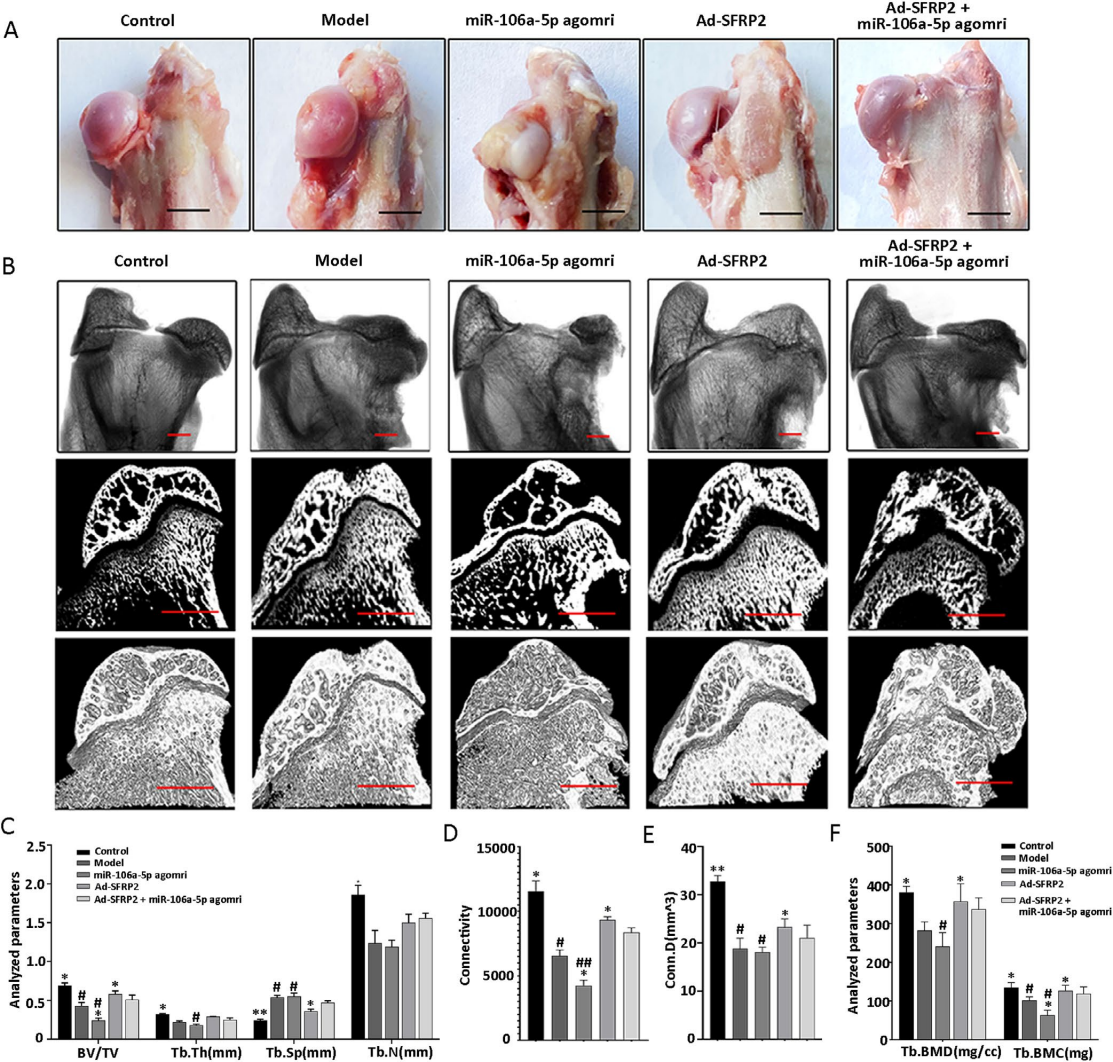
SFRP2 accelerates bone regeneration and reduces deformity of the defective femoral head in vivo. (A) Macroscopic morphological image of the femoral head in the control, model, miR‐106a‐5p agomir, Ad‐SFRP2, and Ad‐SFRP2 + miR‐106a‐5p agomir groups. (B–F) Representative images of micro‐CT and quantitative analysis of the parameters. Scale bars: 10 mM. Results are shown as means ± SD for three independent experiments. *p < 0.05; **p < 0.01 versus the model group; ^#^ *p* < 0.05; ^##^ *p* < 0.01 versus Ad‐SFRP2 group + miR‐106a‐5p agomir.

**FIGURE 6:**
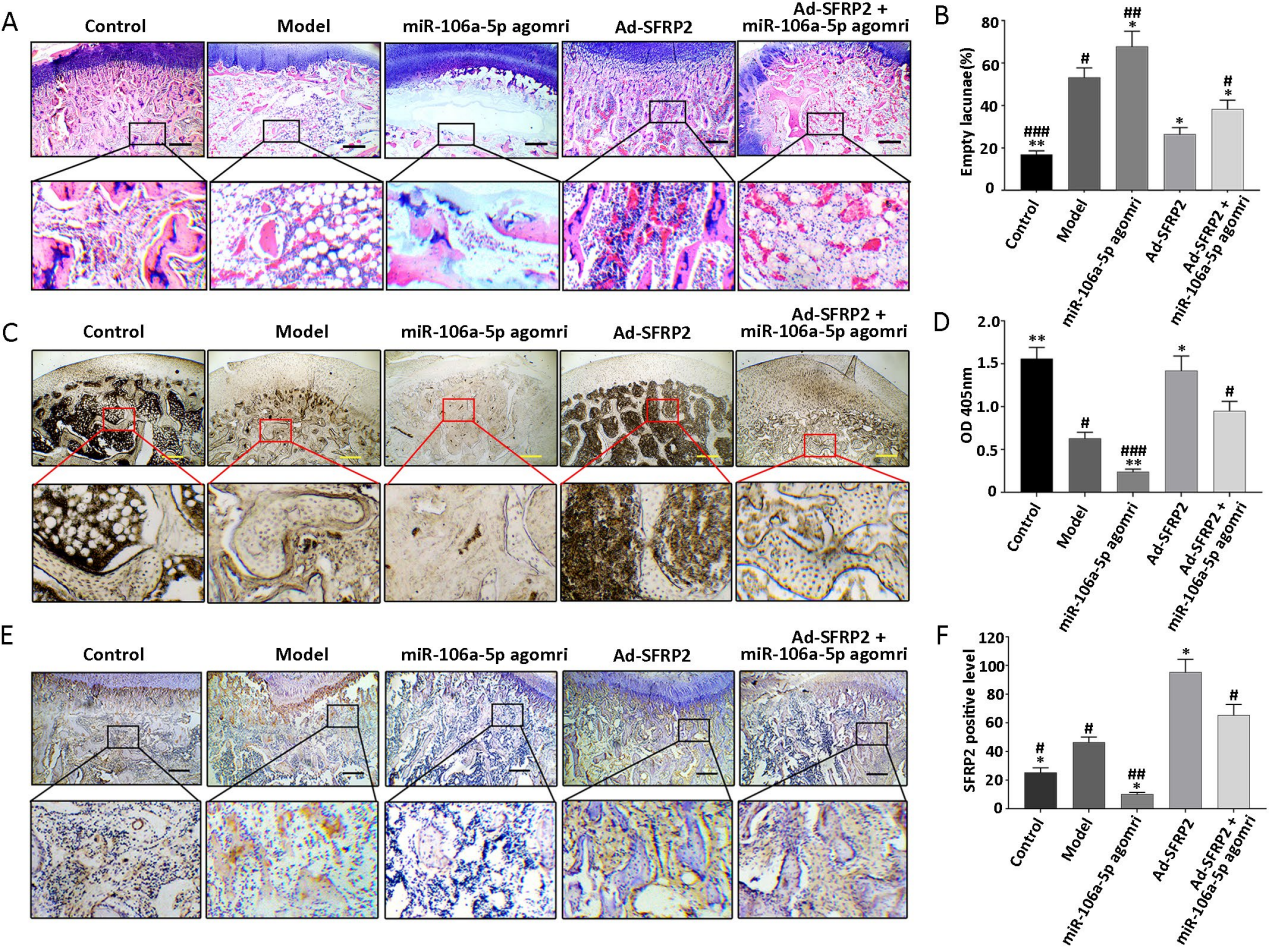
SFRP2 promotes cell proliferation and enhances cell osteogenic differentiation in vivo. (A, B) Haematoxylin and eosin staining shows empty bone lacunae in the femoral head and its proportions. (C, D) ALP staining demonstrating osteogenic activity (and quantitative analysis results). (E, F) Representative image of immunohistochemical staining with proportion of SFRP2‐positive cells. Results are shown as means ± SD for three independent experiments. *p < 0.05; **p < 0.01 versus model group; ^#^ *p* < 0.05; ^##^ *p* < 0.01; ^###^ *p* < 0.001 versus Ad‐SFRP2 + miR‐106a‐5p agomir group.

The defective femoral head of the rabbit model was treated with Ad‐SFRP2 and miR‐106a‐5p agomir to further validate the results obtained in vitro. Macroscopic morphometrical observation of the defective femoral head demonstrated typical manifestations, which were similar to those observed in the model group. In the Ad‐SFRP2 group, the femoral head demonstrated no signal collapse and a good spherical structure; the surface of the cartilage was also smooth and glossy (Figure 5A). The deformity of the femoral head had reduced significantly, and micro‐CT analyses demonstrated a significant improvement in bone volume/total volume, bone mineral density of trabecular bone, bone mineral content of trabecular bone, connectivity and connectivity (Figure 5B–F). Evaluation after haematoxylin and eosin staining showed the proportion of empty bone lacunae to have significantly decreased (Figure 6A,B), and ALP staining showed an obvious increase in activity (Figure 6C,D). Immunohistochemical staining showed that the number of SFRP2‐positive cells had increased significantly in the Ad‐SFRP2 group compared with the model and miR‐106a‐5p agomir groups (Figure 6E,F). Notably, SFRP2 could rescue the inhibitory effect of miR‐106a‐5p on osteogenic differentiation in the Ad‐SFRP2 + miR‐106a‐5p agomir group (Figures 5 and 6). These results confirmed the role of SFRP2 in accelerating bone regeneration and reducing femoral head deformity in the rabbit model of Perthes disease.

## 4 Discussion

The pathogenesis and mechanisms of bone regeneration in Perthes disease remain unclear [5]. As clinical specimens of the defective femoral head are difficult to obtain, animal models offer the best alternative for studying the underlying mechanisms. A previous study that used RNA‐seq showed differential expression of SFRP2 and miR‐106a‐5p in the repaired femoral head of an immature rabbit model [11]. The findings from the present study demonstrated the novel role of the miR‐106a‐5p/SFRP2 axis in the regulation of osteogenic differentiation in osteoblast precursors and bone regeneration.

The SFRP family consists of mammalian secreted glycoproteins, and each member performs various biological functions in different tissues via different mechanisms; this includes the growth and development of articulation surfaces and the occurrence and progression of various diseases [17, 18]. SFRP2, which regulates the pathological processes of many diseases, is an important member of the SFRP family [19, 20]. In this context, Morello and Ikegawa et al. suggested that SFRP2 is significantly upregulated during limb development, and that SFRP2 deficiency may result in syndactylism and brachydactylism [8, 9]. In their study, Lin et al. transfected BMSCs with the IGF‐1 vector; they found that SFRP2 increased the activity and proliferation ability of BMSCs, inhibited apoptosis, and improved repair and regeneration ability after myocardial infarction [21]. In their study, de Castro et al. also found that knockout of the mouse SFRP2 gene significantly inhibited in vitro osteogenic differentiation in BMSCs and SSCs [10]. Cho et al. confirmed that SFRP2 can promote osteogenic differentiation of mouse BMSCs in vitro. It could also significantly enhance β‐glycerophosphate and ascorbic acid‐induced ALP activity and activate Wnt/β‐catenin/TCF conduction [22]. However, certain studies have reported conflicting results. In their study, Kim and Sathi et al. found that the SFRP2 secreted by multiple myeloma cells inhibited differentiation and bone formation in osteogenic precursor cells via the Wnt/β‐catenin pathway [23, 24]. In this context, SFRP2 is considered to be an important upstream regulator of the Wnt/β‐catenin pathway [7, 25], which plays an important role in bone development and bone homeostasis [26]. We therefore further explored the relationship between SFRP2 and the Wnt/β‐catenin pathway. Consistent with findings from previous studies, our results demonstrated that SFRP2 can promote proliferation of BMSCs and MC3T3‐E1 cells, inhibit apoptosis, enhance osteogenic differentiation via the Wnt/β‐catenin pathway, accelerate bone regeneration, and reduce the deformity of the defective femoral head.

Osteogenic differentiation and bone regeneration are long‐term and complex biological processes, that are also regulated by epigenetic factors [27, 28]. The miRNAs are widely involved in bone growth and the progression of osteoarthropathy (such as osteoporosis and osteonecrosis) [12, 29]. In recent years, miR‐106a‐5p has been demonstrated to play an important role in the regulation of osteochondral development and maintenance of bone homeostasis. In their study, Wu et al. found that exosomes containing osteoclast‐derived miR‐106a‐5p can enhance osteogenic differentiation in BMSCs and accelerate repair of bone defects [14]. However, Hui et al. screened microRNA differential expression profiles of BMSCs in adolescent patients with idiopathic scoliosis, and found miR‐106a‐5p to be significantly upregulated; they speculated that miR‐106a‐5p has an osteogenic inhibitory effect, which is associated with bone mass loss [15]. Peltzer et al. also suggested that BMSC‐derived miR‐106a‐5p have an inhibitory effect on osteogenesis under the influence of interferon‐γ [30]. In addition, Xiang et al. found that miR‐106a‐5p inhibited the osteogenic differentiation and mineralisation of human periodontal ligament stem cells in an osteogenic medium [31]. In this study, we injected Ad‐SFRP2 and miR‐106a‐5p agomir into the subchondral bone region of the femoral head. This location was selected to maximise direct delivery to the osteogenic progenitor cells in the bone marrow microenvironment, which are critical for bone regeneration. In agreement with these findings, our results indicated that miR‐106a‐5p reduced proliferation of osteoblast precursors, increased apoptosis, inhibited osteogenic differentiation in vitro, and delayed restorative bone regeneration in defective femoral heads in vivo. We also confirmed miR‐106a‐5p to have a negative regulatory effect on SFRP2, and verified the target binding site of miR‐106a‐5p and SFRP2 mRNA‐3′UTR at the molecular level.

This study utilised a surgically induced femoral head defect model to simulate bone regeneration processes. While this model shares pathological features with the repair phase of Perthes disease, it does not fully recapitulate the multifactorial aetiology (e.g., vascular insufficiency) of clinical Perthes disease. Further validation in disease‐specific models is warranted.

## 5 Conclusion

In summary, our findings demonstrate that the miR‐106a‐5p/SFRP2 axis activation enhances osteogenesis and bone regeneration in femoral head defects. While further studies are needed to validate its role in clinical Perthes disease, this mechanism may represent a therapeutic target for bone repair in conditions with analogous pathophysiology, including the regenerative phase of Perthes disease. Further studies are needed to explore the exact mechanism of action of SFRP2 and the clinical use of miR‐106a‐5p/SFRP2 for the repair of the defective femoral head.

## Author Contributions

**Tianjiu Zhang:** conceptualization (lead), data curation (equal), investigation (equal), visualization (equal), writing – original draft (lead). **Jiafei Yang:** data curation (supporting), formal analysis (equal), software (equal), visualization (equal). **Song Yu:** funding acquisition (lead), methodology (lead), supervision (lead), writing – review and editing (lead).

## Conflicts of Interest

The authors declare no conflicts of interest.

## Supporting information


Figure S1.

## Data Availability

The raw data supporting the conclusions of this article will be made available by the authors on request.
