# Supplementary figures and images for: Secreted Frizzled‐Related Protein 2 Promotes Osteogenic Differentiation and Bone Regeneration in Perthes Disease When Targeted by miR‐106a‐5p
Source: J Cell Mol Med. 2025 Sep 21;29(18):e70804. doi: 10.1111/jcmm.70804 (PMC12450604; doi:10.1111/jcmm.70804)

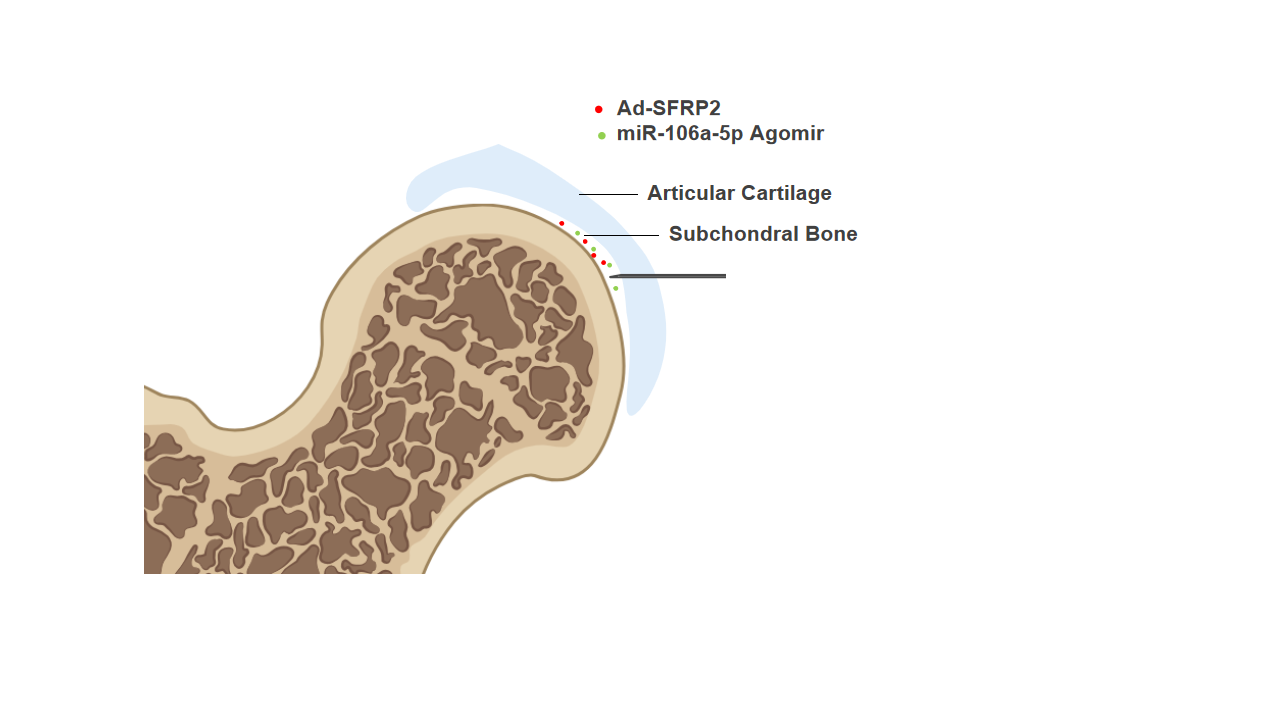

Supplement: Supplementary file 1 — Figure S1. [file JCMM-29-e70804-s001.tif]
